# Supplementary material for: Content-rich biological network constructed by mining PubMed abstracts
Source: BMC Bioinformatics. 2004 Oct 8;5:147. doi: 10.1186/1471-2105-5-147 (PMC528731; doi:10.1186/1471-2105-5-147)
Supplement: Additional File 2 — The original results of the above study (non-essential files are deleted to keep the file size under the limit set by BMC bioinformatics). [file 1471-2105-5-147-S2.bz2 › chilibotAdditionalFile2/dip30/614ID10619841E10997/html/GTF2B_TBP.html]

 


 **GTF2B** and **TBP** 
  
Found 354 abstracts in PubMed, retrieved 30.  
 

 What does Google say? 
 PDF only 
| .edu only 

---

**Interactive relationship** (e.g. stimulation, inhibition, etc)

**Inhibitory relationship**- TFIIB  [ **GTF2B** ]  K272I was weakly suppressed by  **TBP**  mutants in which K239 was changed to hydrophobic residues.  Ref: 12446790 Mol Cell Biol, 2002
- We also found through chromatin immunoprecipitation experiments that the interaction between NZFP and  **TBP**  inhibits binding of TFIIA and TFIIB  [ **GTF2B** ] .  Ref: 12788093 Biochem Biophys Res Commun, 2003
- TFIIB  [ **GTF2B** ]  with the G204D mutation TFIIB  [ **GTF2B** ]  G204D was suppressed by hydrophobic substitutions at lysine 239 of  **TBP** .  Ref: 12446790 Mol Cell Biol, 2002
- NC2 binds to the TATA binding protein  [ **TBP** ]   **TBP** , blocking the recruitment of TFIIA and TFIIB  [ **GTF2B** ] , and thereby inhibiting preinitiation complex assembly.  Ref: 12237409 Proc Natl Acad Sci U S A, 2002
- TFIIB  [ **GTF2B** ]  greatly accelerates formation of a bent TFIIB  [ **GTF2B** ]   **TBP**  TATA box complex, and the inhibitory DNA binding surface of  **TBP**  contributes to the cooperativity of binding to TFIIB  [ **GTF2B** ] .  Ref: 11893333 Cell, 2002
- A novel  **TBP**  interacting zinc finger protein represses transcription by inhibiting the recruitment of TFIIA and TFIIB  [ **GTF2B** ] .  Ref: 12788093 Biochem Biophys Res Commun, 2003
- However, this mutant TFIIB  [ **GTF2B** ]  was strongly suppressed by conservative substitutions in the DNA binding surface of  **TBP** .  Ref: 12446790 Mol Cell Biol, 2002
**Neutral relationship**- The interacting region identified in IE62 overlaps with or borders on sites involved in interactions with the VZV IE4 protein and the cellular factors  **TBP**  and TFIIB  [ **GTF2B** ] .  Ref: 12855699 J Biol Chem< MedlineTA>J Biol Chem, 2002
- We used a rice whole cell extract in vitro transcription system to characterize the functional interactions of recombinant plant  **TBP**  and TFIIB  [ **GTF2B** ] .  Ref: 11971135 Plant Cell, 2002
- We found that it binds purified  **TBP**  and TFIIB  [ **GTF2B** ]  in gel mobility shift assays with cooperative enhancement of binding affinity.  Ref: 11943774 J Biol Chem, 2002
- A second region on the stirrup of the C terminal  **TBP**  repeat corresponds to the TFIIB  [ **GTF2B** ]  binding surface.  Ref: 11909971 Mol Cell Biol, 2002
- Interdependent interactions between TFIIB  [ **GTF2B** ] , TATA binding protein  [ **TBP** ] , and DNA.  Ref: 12446790 Mol Cell Biol, 2002
- The typical  **TBP**  repeat signature and the amino acid motives involved in TFIIA and TFIIB  [ **GTF2B** ]  interactions were conserved in this new  **TBP**  like protein.  Ref: 12154093 J Biol Chem, 2002
- This is accomplished by the ability of the TFIIIB subunit, TFIIB  [ **GTF2B** ]  related factor Brf1, to make stable protein protein interactions with TATA binding protein  [ **TBP** ]   **TBP**  and place it on the promoter less upstream DNA.  Ref: 12682361 Nucleic Acids Res, 2003
- The fission yeast TFIIB  [ **GTF2B** ]  related factor limits RNA polymerase III to a TATA dependent pathway of  **TBP**  recruitment.  Ref: 12682361 Nucleic Acids Res, 2003
- A shared surface of  **TBP**  directs RNA polymerase II and III transcription via association with different TFIIB  [ **GTF2B** ]  family members.  Ref: 12535529 Mol Cell, 2003
- Interactions of a DNA bound transcriptional activator with the  **TBP**  TFIIA TFIIB  [ **GTF2B** ]  promoter quaternary complex.  Ref: 12538582 J Biol Chem, 2003
- Interaction of the equine herpesvirus 1 EICP0 protein with the immediate early IE protein, TFIIB  [ **GTF2B** ] , and  **TBP**  may mediate the antagonism between the IE and EICP0 proteins.  Ref: 12552007 J Virol, 2003
- Notably, the topology of the  **TBP**  TFIIA TFIIB  [ **GTF2B** ]  promoter complex is not altered significantly by the interaction with DNA bound activators.  Ref: 12538582 J Biol Chem, 2003
- In vitro interaction assays revealed that the EICP0 protein interacted directly with the basal transcription factors TFIIB  [ **GTF2B** ]  and  **TBP** .  Ref: 12552007 J Virol, 2003
- Taken together, our results indicate that the spatial positioning of the DNA bound activation domain is important for efficient activation, possibly by maximizing its interactions with the transcriptional machinery including the  **TBP**  TFIIA TFIIB  [ **GTF2B** ]  promoter quaternary complex.  Ref: 12538582 J Biol Chem, 2003
- This result implies DNA bending and looping of promoter DNA as a result of the physical interaction between GAL4 VP16 and an interface of the  **TBP**  TFIIA TFIIB  [ **GTF2B** ]  complex.  Ref: 12538582 J Biol Chem, 2003
- This protein protein interaction on promoter DNA minimally requires the presence of one GAL4 binding site and the formation of a quaternary complex containing  **TBP** , TFIIB  [ **GTF2B** ] , and TFIIA on the TATA element.  Ref: 12538582 J Biol Chem, 2003
- Taken together, these results suggested that interaction of the EICP0 protein with the IE protein, TFIIB  [ **GTF2B** ] , and  **TBP**  may mediate the antagonism between the IE and EICP0 proteins.  Ref: 12552007 J Virol, 2003
- Second, unlike yeast  **TBP** , human  **TBP**  can use a shared surface to interact with two different TFIIB  [ **GTF2B** ]  family members TFIIB  [ **GTF2B** ]  and Brf2 to initiate transcription by different RNA polymerases.  Ref: 12535529 Mol Cell, 2003
- The TBPs with increased affinity could not suppress TFIIB  [ **GTF2B** ]  G204D, leading us to propose a two step model for the interaction between TFIIB  [ **GTF2B** ]  and the  **TBP**  DNA complex.  Ref: 12446790 Mol Cell Biol, 2002
- Our simulated TATA  **TBP**  complexes develop sequence dependent structure and motion trends that may lead to favorable orientations for high activity variants with respect to binding TFIIA, TFIIB  [ **GTF2B** ] , and other transcription factors, while conversely, accelerate dissociation of low activity TATA  **TBP**  complexes.  Ref: 12767124 Biopolymers, 2003
- In interphase cells, GFP  **TBP**  distributes throughout the nucleoplasm and shows a FRAP that is 100 fold slower than the general transcription factor GFP TFIIB  [ **GTF2B** ] .  Ref: 11809839 Mol Biol Cell, 2002
- Rice TATA binding protein  [ **TBP** ]  interacts functionally with transcription factor IIB  [ **GTF2B** ]  and the RF2a bZIP transcriptional activator in an enhanced plant in vitro transcription system.  Ref: 11971135 Plant Cell, 2002
- These data strongly suggest that the repression by NZFP occurs through its binding to both DNA and  **TBP**  and the resulting NZFP  **TBP**  promoter complex inhibits preinitiation complex assembly by preventing binding of TFIIA and TFIIB  [ **GTF2B** ] .  Ref: 12788093 Biochem Biophys Res Commun, 2003

**Non-interactive relationship** (e.g. studied together, co-existance, homology, etc.)

- The inhibitory effect of E1A 13S on the assembly of the PIC is overcome when excess  **TBP**  is added back in the reaction, while addition of either excess TFIIA or TFIIB  [ **GTF2B** ]  were ineffective.  Ref: 12818428 Biochim Biophys Acta, 2003
- Transcription factor IIIB TFIIIB, consisting of the TATA binding protein  [ **TBP** ]   **TBP** , TFIIB  [ **GTF2B** ]  related factor Brf1 and Bdp1, is a central component in basal and regulated transcription by RNA polymerase III.  Ref: 12660736 Nature, 2003
- Likewise, in stressed cells  **TBP**  transcription factor B TFB TFIIB  [ **GTF2B** ]  association was increased by comparison with unstressed controls.  Ref: 11814691 Gene, 2002
- In addition, EMSA studies show that adenovirus E1A 13S oncoprotein is unable to disrupt either the preestablished TFIIA  **TBP**  TATA or TFIIA TFIIB  [ **GTF2B** ]   **TBP**  TATA complexes, but does inhibit complex formation when all transcription factors were simultaneously added.  Ref: 12818428 Biochim Biophys Acta, 2003
- The  **TBP**  gene was mutagenized, and alleles that suppress the slow growth phenotypes of the TFIIB  [ **GTF2B** ]  mutants were isolated.  Ref: 12446790 Mol Cell Biol, 2002
- Archaea have a eukaryotic type of transcriptional machinery containing homologues of the transcription factors TATA binding protein  [ **TBP** ]   **TBP**  and TFIIB  [ **GTF2B** ]  TFB and a pol II type of RNA polymerase.  Ref: 12381724 J Biol Chem, 2003
- Using  **TBP**  and TFIIB  [ **GTF2B** ] , we show that  **TBP**  can bind the TATA box through a regulated two step mechanism, involving a transition from unbent complex to bent complex.  Ref: 11893333 Cell, 2002
- TATA binding protein  [ **TBP** ]   **TBP**  and transcription factor IIB  [ **GTF2B** ]  TFIIB  [ **GTF2B** ]  are key factors for the assembly of eukaryotic transcription initiation complexes.  Ref: 11971135 Plant Cell, 2002
- These changes led to increased affinity between  **TBP**  and TFIIB  [ **GTF2B** ] .  Ref: 12446790 Mol Cell Biol, 2002
- Native human TATA binding protein  [ **TBP** ]  simultaneously binds and bends promoter DNA without a slow isomerization step or TFIIB  [ **GTF2B** ]  requirement.  Ref: 12791683 J Biol Chem< MedlineTA>J Biol Chem, 2002
- We show that TFIID  [ **TBP** ]  and TFIIB  [ **GTF2B** ]  can remain associated with active gene promoters during mitosis whereas RNA polymerase II is displaced, and also that NC2, originally identified as ubiquitous repressor of transcription, is associated with active gene promoters in asynchronous cell populations and is displaced from some, but not all, genes in mitotic cells.  Ref: 11744923 Nat Cell Biol, 2002
- To examine the roles of the enhanceosome and CIITA in these processes, we analysed the level of promoter associated hyperacetylated histones H3 and H4,  **TBP** , TFIIB  [ **GTF2B** ]  and RNA poly merase II in cells lacking RFX or CIITA.  Ref: 11889043 EMBO J, 2002
- Here we used chromatin immunoprecipitation ChIP assays to examine the association of TFIID  [ **TBP** ] , TFIIB  [ **GTF2B** ] , NC2 and RNAP II with various gene promoters in asynchronous and mitotic human cell populations.  Ref: 11744923 Nat Cell Biol, 2002
- Site specific protein DNA photo cross linking was used to show that, when bound to its cognate site at various distances upstream of the TATA element, the chimeric transcriptional activator GAL4 VP16 can physically interact with a TATA box binding protein  [ **TBP** ]   **TBP**  transcription factor IIA TFIIA TFIIB  [ **GTF2B** ]  complex assembled on the TATA element.  Ref: 12538582 J Biol Chem, 2003
- The amino terminal half of Brf1 shares a high degree of sequence similarity with the polymerase II general transcription factor TFIIB  [ **GTF2B** ] , but it is the carboxy terminal half of Brf1 that contributes most of its binding affinity with  **TBP** .  Ref: 12660736 Nature, 2003
- We show that the VP16 activation domain directly interacts with TATA binding protein  [ **TBP** ]   **TBP** , TFIIB  [ **GTF2B** ] , and the SAGA histone acetylase complex in vivo.  Ref: 12297514 J Biol Chem, 2002
- Our unpublished data showed that the IE protein interacts with the same domain aa 125 to 174 of TFIIB  [ **GTF2B** ]  and with  **TBP** .  Ref: 12552007 J Virol, 2003
- Chromatin immunoprecipitation analyses demonstrated that T cell activation triggers in vivo binding of endogenous c Rel, c Jun, C EBP beta, and HMG IC to the bfl 1 regulatory region, coincident with selective recruitment of coactivators TAFII250 and p300, SWI SNF chromatin remodeling factor component BRG 1, and basal transcription factors TATA binding protein  [ **TBP** ]   **TBP**  and TFIIB  [ **GTF2B** ] , as well as hyperacetylation of histones H3 and H4.  Ref: 12665576 Mol Cell Biol, 2003
- One mutation G204D results in disruption of a protein protein contact between TFIIB  [ **GTF2B** ]  and TATA binding protein  [ **TBP** ]   **TBP** , while the other K272I disrupts an interaction between TFIIB  [ **GTF2B** ]  and DNA.  Ref: 12446790 Mol Cell Biol, 2002
- Finally, we show that CtIP and CtBP can interact with the general transcription factors, TATA binding protein  [ **TBP** ]  and transcription factor IIB  [ **GTF2B** ] , which suggests a possible mechanism for their deacetylase independent mode of repression.  Ref: 11959865 J Biol Chem, 2002
